# Homogenate-assisted Vacuum-powered Bubble Extraction of Moso Bamboo Flavonoids for On-line Scavenging Free Radical Capacity Analysis

**DOI:** 10.3390/molecules22071156

**Published:** 2017-07-11

**Authors:** Yinnan Sun, Kui Yang, Qin Cao, Jinde Sun, Yu Xia, Yinhang Wang, Wei Li, Chunhui Ma, Shouxin Liu

**Affiliations:** College of Material Science and Engineering, Northeast Forestry University, Harbin 150040, China; sunyinnan1@163.com (Y.S.); nefuyangkui@163.com (K.Y); nefucaoqin@163.com (Q.C.); nefusunjinde@163.com (J.S.); xiayu0712@163.com (Y.X.); nefuwangyinhang@163.com (Y.W.); liwei820927@nefu.edu.cn (W.L.)

**Keywords:** *Phyllostachys pubescens (P. pubescens)*, flavonoids, homogenate-assisted vacuum-powered bubble extraction (HVBE), HPLC, on-line antioxidant capacity

## Abstract

A homogenate-assisted vacuum-powered bubble extraction (HVBE) method using ethanol was applied for extraction of flavonoids from *Phyllostachys pubescens (P. pubescens)* leaves. The mechanisms of homogenate-assisted extraction and vacuum-powered bubble generation were discussed in detail. Furthermore, a method for the rapid determination of flavonoids by HPLC was established. HVBE followed by HPLC was successfully applied for the extraction and quantification of four flavonoids in *P. pubescens*, including orientin, isoorientin, vitexin, and isovitexin. This method provides a fast and effective means for the preparation and determination of plant active components. Moreover, the on-line antioxidant capacity, including scavenging positive ion and negative ion free radical capacity of different fractions from the bamboo flavonoid extract was evaluated. Results showed that the scavenging DPPH˙ free radical capacity of vitexin and isovitexin was larger than that of isoorientin and orientin. On the contrary, the scavenging ABTS^+^˙ free radical capacity of isoorientin and orientin was larger than that of vitexin and isovitexin.

## 1. Introduction

Moso bamboo (*Phyllostachys pubescens*), which belongs to the Gramineae family, is an important bamboo species in Korea, Japan, and China [1]. China has more than 5 million ha of bamboo forests, with Moso bamboo forests making up 63% of the total [2]. Moso bamboo possesses characteristics of high biomass, high growth rate, easy cultivation, extensive competitive ability [3], and it grows widely in the subtropical zone in Asia [4]. The edible shoot of Moso bamboo is a popular delicacy, and the timber of Moso bamboo has been widely used for floor decoration, furniture, and charcoal making [5]. Because of its high economic return, the area under Moso bamboo cultivation has dramatically increased over the past several decades [4].

However, every year, a great amount of the bamboo leaves must be disposed of as waste. Bamboo leaves, which are rich in flavonoids, are considered to be a natural source of antioxidants [6] without toxicity [7,8]. Flavonoids and flavone C-glucosides, a large category of plant polyphenol secondary metabolites, are widely distributed in bamboo leaves [9]. Previous studies have generated particular interest with regard to the beneficial human health effects of bamboo leaves, including their antioxidant [10], antiobesity [11], antitumor [12], and antimicrobial activities [13].

Recently, an on-line technique that coupled high-performance liquid chromatography (HPLC) and post-column detection of the radical scavenging capacity of natural products was reported in the literature [14,15]. It was shown to be a sensitive, selective, and relatively simple method for antioxidant capacity analysis, and has been applied for quick screening of various plant extracts [16,17]. Although Kim et al. evaluated the antioxidant activity of bamboo phenolic compounds by on-line ABTS^+^˙ detection [18], the on-line detection of scavenging free radicals (DPPH˙, ABTS^+^˙) from the fractionation of a bamboo flavonoid extract was not investigated.

The vacuum-powered bubble extraction method is a type of gas-liquid-solid three phase extraction technology based on the generation, growth, and collapse theory of air bubbles in the liquid phase. Using the power produced by the vacuum, bubbles are sparged at the bottom of the equipment into either the liquid phase or a liquid-solid suspension. The bubble-assisted solvent extraction concept was first described by Chen et al. [19]. Compared with conventional solvent extraction, in bubble extraction, a high contact area can be created using less solvent and the natural buoyancy provided by the air core to promote phase separation [20]. Furthermore, the vacuum-powered bubble extraction has the advantages of simple equipment, low energy consumption, simple operation, easy parameter control, and facilitate the scale production. Thus it has been applied in the extraction field [21,22]. Homogenate-assisted extraction is a physical milling method, which facilitates the release of chemical components from materials into the solvent using high-velocity mechanical shearing, stirring, fluid cutting action, and smashing without extra heating or pressure [23]. The homogenate process possesses obvious effects of pulverization and extraction. Homogenate-assisted extraction has been successfully introduced for the extraction of alkaloids, isoflavones, and pigments [24,25].

The purpose of this investigation was to extract flavonoids from bamboo leaves using homogenate-assisted and vacuum-powered bubble extraction (HVBE) technology. The novelty of our study is that the mechanism of vacuum-powered bubble extraction have been interpreted in-depth from the angle of the bubbles generation, growth, and collapsed, in combination with the extraction process, including penetration, dissolution, distribution, and diffusion. The other novelty of this manuscript is the RP-HPLC on-line antioxidant method for detecting four individual flavonoids simultaneously that was set up for detection the radical scavenging capacity of bamboo flavonoids.

## 2. Results and Discussion

### 2.1. Single Factor HVBE Experiments 

#### 2.1.1. Effect of the Fraction of Ethanol on the Homogenate Pretreatment

**Figure 1:**
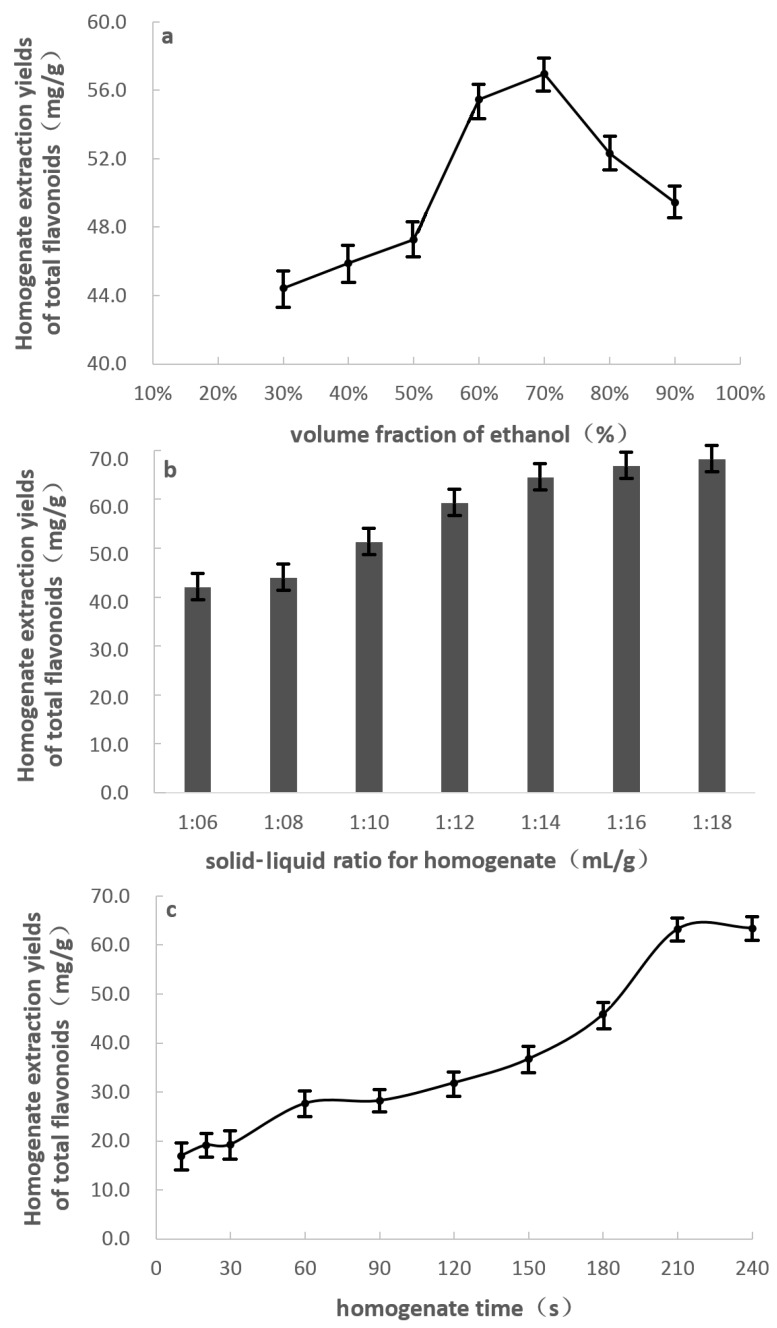
Effect of the ethanol fraction (**a**); solid–liquid ratio (**b**); and the extraction time (**c**) on the homogenate extraction yield of bamboo flavonoids.

The higher the fraction of ethanol was in the solution, the better the permeability of the extraction solvent. According to “like dissolves like” principle, using the total flavonoid content as the evaluation index, at solid–liquid ratios 1:10 mL/g, and homogenate pretreatment 4.0 min, as shown in Figure 1a, the extraction yield of the total flavonoids increased as the fraction of ethanol in solution increased, and 70% ethanol solution was the most effective at extracting the bamboo flavonoid compounds to give an extraction yield of 57.0 ± 1.4 mg/g of total flavonoids. 

#### 2.1.2. Effect of Solid–Liquid Ratio on the Homogenate Pretreatment

A higher solvent content may require complex procedures, produce unnecessary waste, and increase the energy consumption during recycling, while a lower solvent content may lead to incomplete extraction. With homogenate pretreatment 4.0 min, Figure 1b clearly shows that the total flavonoid extraction yield increased with an increase in solvent volume, but at solid–liquid ratios of more than 1:14 (the yield of total flavonoids was 66.2 ± 1.8 mg/g with 70% ethanol solution), it was not significantly influenced by a further increase in the amount of solvent. Therefore, considering the yield of total flavonoids and the energy consumption of solvent recovery, a 1:14 solid–liquid ratio was selected and used in the homogenate-assisted extraction and vacuum-powered bubble extraction studies.

#### 2.1.3. Effect of Extraction Time on Homogenate Pretreatment 

Particle size is a key variable that governs the effectiveness of the homogenate pretreatment because the permeation of the solvent is influenced by the particle diameter. The particle size depends on the homogenate-assisted extraction time. Adding 70% ethanol solution at solid–liquid ratios 1:14 mL/g, Figure 1c shows that as the extraction time increased, the total flavonoid yield initially increased. Using a homogenate extraction time of 210 s, the total flavonoid yield increased inconspicuously (the total flavonoid yield was 63.4 ± 1.2 mg/g using 70% ethanol solution). However, too small particle size may lead to a difficult solid-liquid separation after HVBE. Therefore, a 3.5 min homogenate-assisted treatment was selected as the optimal condition for extracting the flavonoid compounds.

#### 2.1.4. Effect of Extraction Time and Air Velocity on Vacuum-powered Bubble Extraction

**Figure 2:**
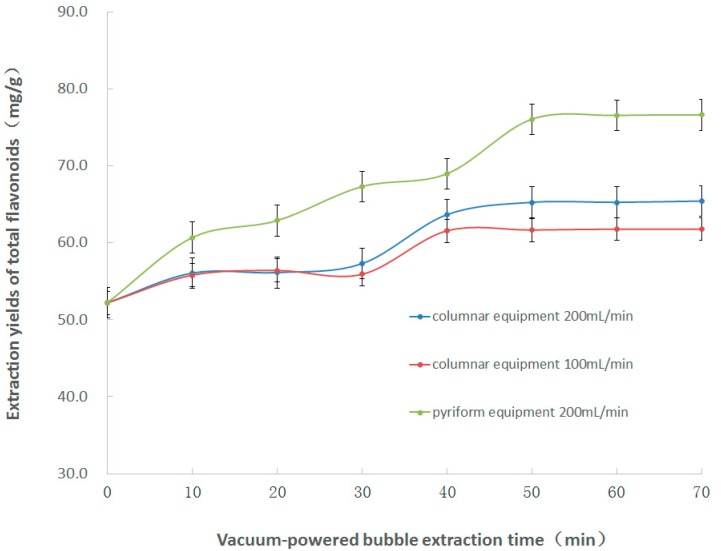
Effect of the extraction time on the vacuum-powered bubble extraction.

As shown in Figure 2, the extractions were carried out at negative pressure using a 200 W vacuum pump for 10, 20, 30, 40, 50, 60, and 70 min. The air velocities for vacuum-powered bubble extraction were 100 and 200 mL/min. The total flavonoid extraction yield increased significantly when the treatment time was increased from 0 to 40 min (the total flavonoid content was 61.6 ± 2.4 mg/g using the 100 mL/min columnar vacuum-powered equipment, and 63.7 ± 2.2 mg/g using the 200 mL/min columnar vacuum-powered equipment). The extraction yield slightly improved as the treatment time was increasing (the total flavonoid content for 45 min was 61.8 ± 2.0 mg/g using the 100 mL/min columnar vacuum-powered equipment, and 65.4 ± 2.0 mg/g using the 200 mL/min columnar vacuum-powered equipment). Therefore, a 200 mL/min air velocity and an extraction time of 40 min were selected for the subsequent experiments.

The vacuum-powered bubble extractions were carried out using different shapes of glass equipment made in house, operated at negative pressure with a 200 W vacuum pump for 10, 20, 30, 40, 50, 60, and 70 min. As shown in Figure 2, at 200 mL/min air velocity, the total flavonoid extraction yield using the pyriform shape device (the total flavonoid content for 50 min was 76.1 ± 2.4 mg/g) was higher than that with the columnar shape device (the total flavonoid content for 50 min was 65.3 ± 2.5 mg/g). The higher air velocity, the amount of bubbles generation was larger. Moreover, in the bottom of pyriform shape device, the bigger specific surface area of the solid wall and solvent interface, the more bubbles were generated. Thus, the total flavonoid extraction yield with the pyriform shape device was higher than that with the columnar shape device.

### 2.2. The Processes Involved in Homogenate Pretreatment

Solvent extraction of natural products from plant cells occurs according to the similar miscibility principle, is the most direct way to obtain bioactive ingredients and avoids the high cost and potentially toxic by-products generated from the chemical synthesis of complex molecules. During homogenate pretreatment, the organic solvent penetrates the cell wall and cell membrane, and if the active ingredient is miscible, it is quickly dissolved. Homogenate extraction pretreatment involves not only the process of pulverizing raw material with mechanical shear force, but also the process of mixing the solid (plant material) and the liquid (extracted solvent). Therefore, it is a type of improved solid-liquid mass transfer process using external force [26].

**Figure 3:**
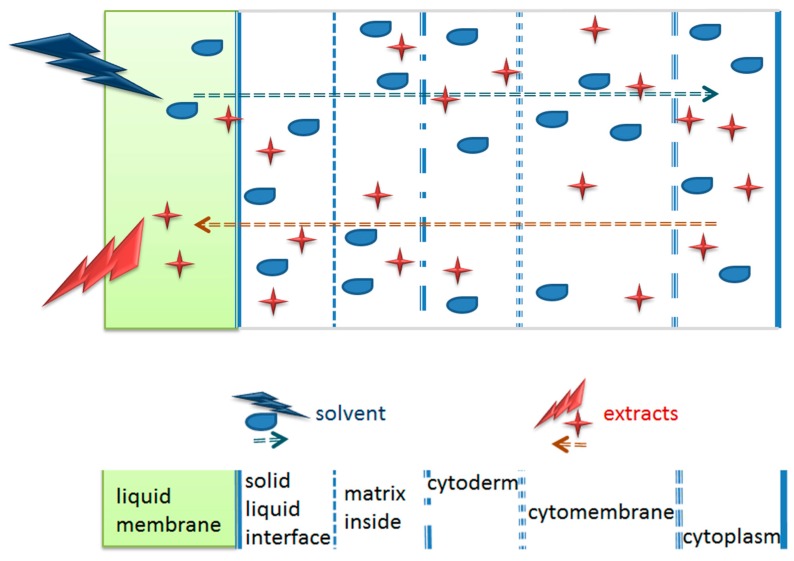
Unstable and non-equilibrium diffusion model of the target component intracellular release.

Diffusion unsteady non-equilibrium model of the target component intracellular release is shown in Figure 3. The microstructure of the plant cell includes three parts, cytoderm, cytomembrane, and cytoplasm. In the solid-liquid system, there is a thinner liquid membrane on the interface of the solid. In the homogenate extraction process, in the presence of mechanical shear force, the solid material particle size decreased, and the smaller the particle size, the bigger the specific surface area of the solid-liquid interface with the solvent turbulent. The rapid replacement of the liquid membrane accelerated the dissolution of the flavonoids from the bamboo leaves. However, the particle size of the bamboo leaves would become too small with a longer homogenate treatment, leading to an increase in the quantity of dissolved impurities.

In summary, homogenate extraction is a type of improved solid-liquid mass transfer process [27], that includes the material crushing step and replacement of the solvent at the solid-liquid interface.

### 2.3. The Influence of the Apparatus Shape on the Generation and Collapse of Air Bubbles

**Figure 4:**
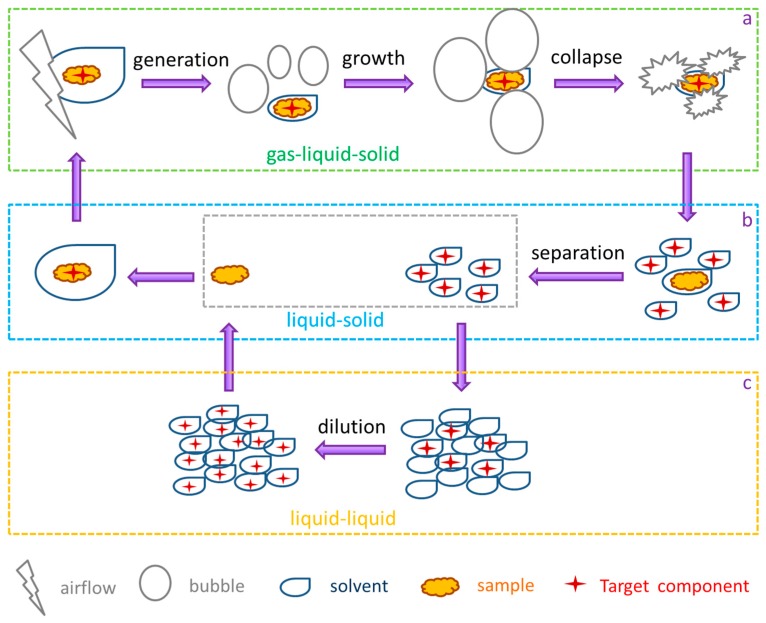
Schematic diagram of the gas–liquid–solid conversion (**a**); the liquid-solid conversion (**b**); and the liquid-liquid conversion (**c**) in the vacuum-powered bubble extraction process.

In the vacuum-powered bubble extraction (VBE) process, the key factor influencing the extraction efficiency of VBE is the behavior of bubbles in solid-liquid phases. Figure 4a shows the generation, growth, and collapse of the bubbles (gas-liquid-solid). Figure 4b depicts the process as the flavonoids are extracted from the bamboo leaves (liquid-solid). Figure 4c shows the concentration gradient generated by solvent turbulence (liquid-liquid). Under vacuum-powered extraction, the bubbles were generated from the solid wall of the glass container. The bigger the specific surface area of the solid wall and solvent interface, the more bubbles were generated. Thus, the quantity of bubbles generated with the pyriform shape device was more than that with the columnar shape device, and the total flavonoid extraction yield for 50 min with the pyriform shape device (76.1 ± 2.4 mg/g) was higher than that with the columnar shape device for 50 min (65.3 ± 2.5 mg/g).

Under negative driving pressure, the air bubbles rose and grew and the larger the negative driving pressure, the faster the bubbles expanded [28]. After expanding, the bubbles collapsed on the surface of the solvent along with the outside lower pressure. As shown in Figure 4a, the bubbles’ collapse resulted in intense cavitation after collision so that the surface of the solid particles was corroded and the solvent diffused into the inside of the solid particles enhancing the intra-particle diffusion. The stronger shear forces from the bubble collapse provided the driving force for enhancing the solid–liquid two-phase mass transfer process. As shown in Figure 4c, the collapse of the bubbles created intensive turbulence and interface effects that resulted in the surrounding liquid drops coalescing into liquid sheets. The liquid sheets containing different solute concentrations rapidly coalesced. As a result, mass transfer took place between the liquid drops [29].

### 2.4 Compared with the Reference Extraction Methods of Flavonoids

For the comparison of the extraction efficiency of HVBE with other reference extraction techniques, SE, RE and UE with 70% ethanol were carried out to extract bamboo flavonoids. The total flavonoid compounds of bamboo leaves obtained by the four methods are also listed in Table 1. The extraction yield of total flavonoids by SE and RE were a little higher (85.6 ± 3.2 and 77.4 ± 2.1 mg/g) than that of HVBE (76.1 ± 2.4 mg/g), and the extraction yield by UE was lowest of all (42.4 ± 1.9 mg/g). Although the extraction yield by SE and RE were higher, the raw material need being grinded and the suspension being heated (90 °C), extraction time was longer (4.0 h) than that of HVBE (1.06 h). In UE process, the suspension needn’t being heated and pressurized, but the extraction yield by UE was lowest of all (42.4 ± 1.9 mg/g). However, in HVBE process, the suspension need being pressurized while needn’t being heated. The advantage of HVBE is shorter extraction time (1.06 h) and the operation needn’t being grinded. Furthermore, the extraction yield by HVBE was also high (76.1 ± 2.4 mg/g).

### 2.5 On-Line Radical Scavenging Capacity of Bamboo Flavonoids

#### 2.5.1. HPLC Quantitative Analysis of Bamboo Flavonoids

**Figure 5:**
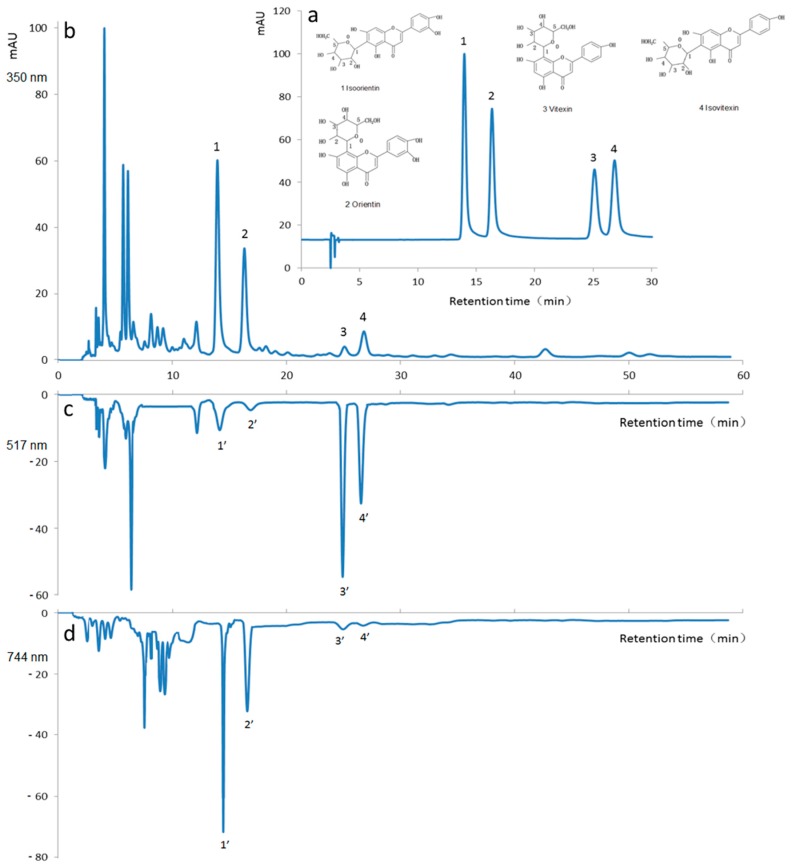
RP-HPLC chromatogram of the flavonoid standards and the on-line scavenging free radical capacity of the *P. pubescens* leaf sample. (**a**) RP-HPLC chromatogram of the bamboo flavonoid standards detected at 350 nm: 1 isovitexin, 2 orientin, 3 vitexin and 4 isoorientin; (**b**) RP-HPLC chromatogram of the *P. pubescens* sample detected at 350 nm; (**c**) On-line scavenging DPPH˙ free radical stability of the *P. pubescens* sample detected at 517 nm; (**d**) On-line scavenging ABTS^+^˙ free radical stability of the *P. pubescens* sample detected at 744 nm.

The purpose of this study was to develop a simple and rapid HPLC method for the simultaneous determination of four active flavonoid components (isoorientin, orientin, vitexin, and isovitexin) in bamboo leaves. When an acetonitrile-2.0% water acetic acid solution (14:86, *v*/*v*) was chosen as the mobile phase, the four analytes of interest were completely separated from the extract of bamboo leaves with good peak shapes using an isocratic elution. Typical chromatograms corresponding to a standard mixture and the chemical structures of the selected four flavonoids (isoorientin, orientin, vitexin, and isovitexin) are shown in Figure 5a, and the chromatogram of the bamboo leaves extract is shown in Figure 5b.

Good sensitivity for all the analytes was observed by monitoring the UV signal at 350 nm. The identities of the eluted peaks were determined by comparing their retention times with those of the standards. The retention time of isoorientin, orientin, vitexin, and isovitexin was 13.97, 16.34, 25.12, and 26.83 min, respectively. And the content of the individual flavonoids in *P. pubescens* leaves samples obtained by HVBE was 123.5 ± 5.2 µg/g, 92.2 ± 1.5 µg/g, 12.5 ± 0.1 µg/g, and 40.5 ± 0.7 µg/g, respectively (Table 2).

#### 2.5.2. On-Line Radical Scavenging Capacity of Bamboo Flavonoids

The purpose of the on-line free radical scavenging experiment was to evaluate the antioxidant activity of the four bamboo flavonoids, including isoorientin, orientin, vitexin, and isovitexin. The on-line radical scavenging capacity of the bamboo leaves extract is shown in Figure 5c (for scavenging DPPH˙ free radical) and Figure 5d (for scavenging ABTS^+^˙ free radical). 

The efficient separation of the individual flavonoid components in bamboo leaves was achieved by using reversed phase HPLC and UV detection at two wavelengths (517 nm for detection of scavenging DPPH˙ free radical, and 744 nm for detection of scavenging ABTS^+^˙ free radical). Flavonoids, a large category of plant polyphenol secondary metabolites are considered to be a natural source of antioxidants [6]. The 7-OH group in flavonoids plays an important role as the site of ionization and of electron transfer according to sequential proton loss electron transfer mechanism [30]. From the antioxidant action viewpoint, the result of this mechanism is the same as in hydrogen atom transfer mechanism to the free radicals. Additional antioxidant activity is assigned to the presence of a hydroxyl group at the 3- and 5-positions. Results showed that the scavenging DPPH˙ of vitexin and isovitexin was larger than that of isoorientin and orientin (Figure 5c). On the contrary, the scavenging ABTS^+^˙ free radical capacity of isoorientin and orientin was larger than that of vitexin and isovitexin (Figure 5d). These active flavonoids possessed an *o*-dihydroxy group in the B-ring, which conferred higher stability in the radical form and participation in electron delocalization. This conclusion was in good agreement with those reported in the previous literature [18]. The on-line method only provided a relatively radical scavenging capacity of individual flavonoid components, and the antioxidant stability of individual bamboo flavonoid components was going to publish in other research paper.

## 3. Materials and Methods

### 3.1. Materials 

#### 3.1.1. Plant Materials

*P. pubescens* leaves as the waste were collected from a bamboo forest in the Yibin area (Sichuan, China) in October 2016. The bamboo leaves were air dried at room temperature in a shady and ventilated place, and then the moisture of the bamboo leaves was measured after drying at a constant temperature of 105 ± 3 °C as 5.99%.

#### 3.1.2. Chemical Materials

Rutin, orientin, isoorientin, vitexin, and isovitexin standards with higher than 98% purity were obtained from the National Institute for the Control of Pharmaceutical and Biological Products (Beijing, China). Deionized water was purified by a Milli-Q water purification system (Millipore, Billerica, MA, USA). HPLC grade acetonitrile and acetic acid were purchased from J&K chemical Ltd. (Shanghai, China). The other chemicals were analytical grade and purchased from Beijing Chemical Reagents Co. (Beijing, China). All solutions were filtered through 0.45-μm membranes (GuangFu Chemical Reagents Co., Tianjin, China) before use for HPLC analysis.

### 3.2. Apparatus

**Figure 6:**
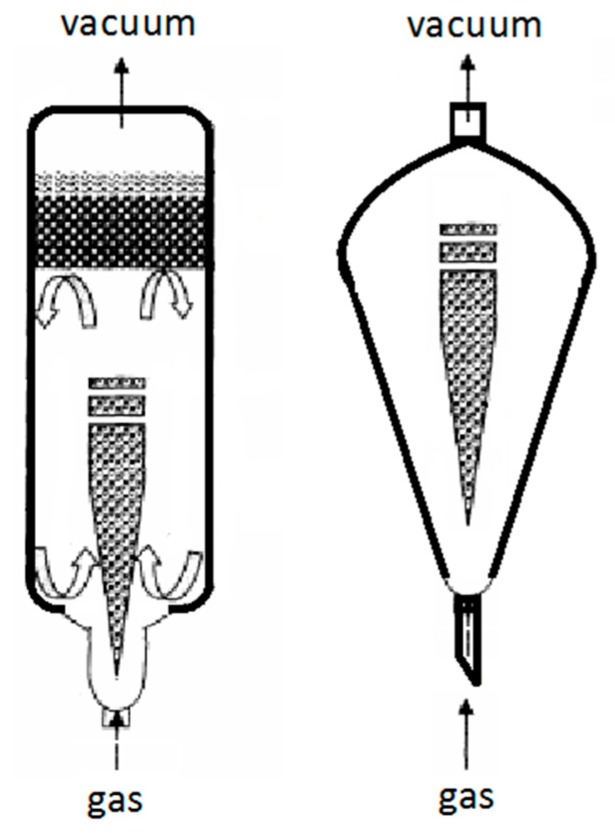
Schematic diagram of the HVBE apparatus.

The HVBE method was applied for extracting the bamboo flavonoids. A 200 W homogenate machine (HANUO-JJ2, Shanghai Hannuo Instrument Co., Ltd., Shanghai, China) was used to grind the material. The vacuum-powered bubble extraction glass columns were made in house, and were connected to a SHB-Ш circulating water vacuum pump (Great Wall Scientific Industry and Trade Co., Ltd., Zhengzhou, China) to control the air flow rate. The shape of the glass columns is shown in Figure 6.

From Figure 6, under the negative pressure drive, the air bubbles are generated from the bottom of the device, and then rise, grow and expand. After expanding, the bubbles collapse on the liquid surface, and the collapse of the bubbles creates intensive turbulence and interface effects. As a result, mass transfer takes place between the solid and liquid phase.

### 3.3. Methods

#### 3.3.1. Detection of Total Flavonoids in *P*. *pubescens* Leaves

Rutin was selected as the standard material for the total flavonoid detection as previously described by Chang et al. [31]. Briefly, diluted sample obtained from HVBE (1.0 mL) was transferred to a 10 mL colorimetric tube, then 5% (*w*/*v*) sodium nitrite (1.0 mL), 10% (*w*/*v*) aluminum chloride (1.0 mL) and 4% (*w*/*v*) sodium hydroxide (6.0 mL) were added in order. The final volume was adjusted to 10 mL with distilled water. The mixture was blended and allowed to stand for 15 min. Then, the absorbance was determined at 500 nm against a water blank using a UV-Vis spectrophotometer (UV-2550, Shimadzu, Tokyo, Japan). The amount of the total flavonoids was expressed as the rutin equivalents in 1.0 g bamboo leaves using the standard calibration curve. A total of 25.0 mg rutin was dissolved in methanol (50 mL, 0.5 mg/mL) as a stock solution. A series of rutin standard solutions in the range of 3.125–50.0 µg/mL were prepared in methanol and a linear response was obtained (*Y* = 8.3201*X* + 0.0065, *R*^2^ = 0.9999) using the above method.

#### 3.3.2. Homogenate-Assisted Vacuum-Powered Bubble Extraction (HVBE) Method

The extraction experiment was performed by adding bamboo leaves material into ethanol solution in the homogenate machine. Dried bamboo leaves sample (0.50 g) were extracted with ethanol–water solution (50 mL, solid:liquid = 1:10 g/mL) by homogenate pretreatment for 3.5 min and filtered. The fractions of ethanol in the ethanol-water solution were 30%, 40%, 50%, 60%, 70%, 80%, and 90% (*v*/*v*). To evaluate the effect of the solid-liquid ratio, 0.50 g of dried bamboo leaves was mixed with 70% ethanol solution as the extraction solvent at a series of different solid-liquid ratios of 1:6, 1:8, 1:10, 1:12, 1:14, 1:16, and 1:18 g/mL. The mixture was placed in the homogenate machine, and the suspension was homogenate extracted for 3.5 min. To investigate the effect of extraction time on the total flavonoid yield using homogenate extraction, 0.50 g of dried sample was mixed with 70% ethanol solution as the extraction solvent (at a solid–liquid ratio of 1:10 g/mL), and then the homogenate was extracted for 10 s, 20 s, 30 s, 60 s, 90 s, 120 s, 150 s, 180 s, 210 s, and 240 s. After homogenate-assisted extraction, the materials and solvent were added into the vacuum-powered bubble extraction glass column. At room temperature, the air velocities were 100 and 200 mL/min with the extraction column of different shapes. Air was supplied through a pump from the bottom of the extraction column and the flow rate was measured by the flow meter. After vacuum-powered bubble extraction the extracts were filtered through a 0.45 µm filter prior to HPLC analysis.

#### 3.3.3. Reference Extraction Methods of Flavonoids

Soxhlet extraction (SE), reflux extraction (RE) are the conventional extraction methods of natural products, and ultrasonic extraction (UE) is the classic assisted extraction method. Extraction process was performed in ultrasonic unit. 5.0 g of grinding dried bamboo leaves was mixed with 50 mL 70% ethanol solution and then soaked for 3.5 h, the suspension was extracted 30 min by UE. Ultrasonic power and solid–liquid ratio were 200 W and 1:10. The conditions of conventional 70% ethanol SE and RE were same as above except extraction 4.0 h at 90 °C. The extract was filtrated through a 0.45 µm filter for content analysis.

#### 3.3.4. On-Line Assay for Evaluation of Antioxidant Activity

##### HPLC Method for Detection of Four Typical Flavonoids

The four typical bamboo flavonoids found in higher concentrations (isoorientin, orientin, vitexin, and isovitexin) were separated from the bamboo extract by HPLC, which improved from the method of Pereira et al. [32]. The HPLC analysis used an HPLC system equipped with a 1525 Bin pump, a 717 automatic column temperature control box and a 2487 UV-detector (Waters, Milford, MA, USA) along with a Waters 717 automatic sample handling system. Chromatographic isolation was conducted on a HiQ sil-C18 reversed-phase column (4.6 mm × 250 mm, 5 µm, KYA TECH Corp., Tokyo, Japan) for the examination of bamboo flavonoids. Isocratic elution was applied during the 60 min run time using a mobile phase of acetonitrile and a 2.0% water acetic acid solution at a ratio of 14:86. The mobile phase flow rate, injection volume, and column temperature were 1.0 mL/min, 5.0 µL and 40 °C, respectively. The four bamboo flavonoids were monitored at 350 nm over the first 30 min. The corresponding calibration curves and linear ranges of detection of the 4 bamboo flavonoids are shown in Table 3.

##### On-Line DPPH˙ Free Radical Scavenging Method

A total of 15.4 mg DPPH˙ was dissolved in 500 mL of the acetonitrile-2.0% water acetic acid (14:86, *v*/*v*) mobile phase, and used in the on-line DPPH˙ free radical scavenging HPLC method as described in HPLC Method for Detection of Four Typical Flavonoids Section. The four bamboo flavonoids were monitored at 517 nm over 30 min.

##### On-Line ABTS^+^˙ Free Radical Scavenging Method

The working solution was prepared by mixing 7.4 mM ABTS^+^˙ solution and 2.6 mM potassium persulfate solution in equal quantities and allowing them to react for 12 h at room temperature in the dark. The solution was then diluted by mixing 10.0 mL ABTS^+^˙ solution with the mobile phase of 500 mL acetonitrile-2.0% water acetic acid (14:86, *v*/*v*) and used in the on-line ABTS^+^˙ free radical scavenging HPLC method as described in HPLC Method for Detection of Four Typical Flavonoids Section. The four bamboo flavonoids were monitored at 744 nm over 30 min.

#### 3.3.5. Statistical Analysis Method

Results of HVBE method are expressed as the mean ± standard deviation (*n* = 3). Statistical significance was determined using Microsoft Excel statistical fractions. Differences of *p* < 0.05 were considered to be significant.

## 4. Conclusions

In summary, homogenate-assisted vacuum-powered bubble extraction (HVBE) was used for extracting flavonoid compounds from *P. pubescens* leaves. The optimum extraction process involved adding dried bamboo leaves to a 70% ethanol solution at a 1:14 solid-liquid ratio for the 3.5 min homogenate pretreatment. This was followed by transfer of the solid material and solvent into the pyriform shape vacuum-powered device for bubble extraction for 50 min using a 200 mL/min air velocity. The total flavonoid extraction yield was 76.1 ± 2.4 mg/g under the optimized conditions. Furthermore, the mechanism of homogenate-assisted extraction and vacuum-powered bubble extraction was described in detail. Four types of bamboo flavonoids, orientin, isoorientin, vitexin, and isovitexin, were detected by RP-HPLC. The optimized HPLC conditions were acetonitrile-2.0% water acetic acid (14:86, *v*/*v*) as the mobile phase at a flow rate of 1.0 mL/min with a 5.0 µL injection volume and 40 °C column temperature. The on-line free radical scavenging detection of four bamboo flavonoids was carried out at 517 nm (for DPPH˙) and 744 nm (for ABTS+˙), over 60 min. The results showed that the scavenging DPPH˙ capacity of vitexin and isovitexin was larger than that of isoorientin and orientin, while the scavenging ABTS+˙ capacity of isoorientin and orientin was larger than that of vitexin and isovitexin.

## Figures and Tables

**Table 1:** Compared with the reference extraction methods of flavonoids.

Reference Method	Grinding	Extraction Temperature (°C)	Extraction Pressure	Extraction Time (h)	Extraction Yield (mg/g)
SE	Need	90	No need	4.0	85.6 ± 3.2
RE	Need	90	No need	4.0	77.4 ± 2.1
UE	Need	Room temperature	No need	3.5 + 0.5	42.4 ± 1.9
HVBE	No need	Room temperature	Needed	0.06 + 1.0	76.1 ± 2.4

**Table 2:** The content of the individual flavonoids in *P. pubescens* leaves samples obtained by HVBE.

Samples	Isoorientin (µg/g)	Orientin (µg/g)	Vitexin (µg/g)	Isovitexin (µg/g)
1	128.7	91.4	12.6	41.2
2	123.5	93.7	12.4	39.9
3	119.0	92.0	12.4	39.8
4	120.2	93.0	12.6	40.6
5	126.3	90.9	12.5	40.8
Average	123.5	92.2	12.5	40.5

**Table 3:** Standard curves and linear ranges of detection of bamboo flavonoids.

Flavonoids	Retention Time/min	Standard curves	*R*^2^	Linear Range/(µg/mL)
Isoorientin	14.0	*Y* = 14936*X* − 0.116	0.9998	1.6–100.5
Orientin	16.3	*Y* = 12473*X* − 0.844	0.9999	1.8–118.0
Vitexin	25.1	*Y* = 8335.6*X* + 4.6702	0.9994	1.5–94.0
Isovitexin	26.8	*Y* = 9770.2*X* + 5.3156	0.9992	1.5–95.0
